# Salmagundi

**Published:** 1885-02

**Authors:** E. G. Betty


### SALMAGUNDI.

EDITED BY E. G. BETTY, D. D. S.

IRONTON, OHIO, January 14th, 1885.

EDITOR SALMAGUNDI :

I have been experimenting with the hydrochlorate of cocaine and submit you some results of same for Salmagundi if you think them worthy of publication :

Experiment 1. Young married lady with three very sensitive crown cavities in lower molars. Adjusted the rubber dam and proceeded to excavate—cavities excruciatingly sensitive. Applied the cocaine, and after a few minutes was able to proceed with two cavities without producing pain in the slightest degree ; the third remained tender to limited extent after repeated applications, but modified so as to admit of thorough preparation with minimum of pain.

Exp. 2. Married lady with teeth of good structure, but exceedingly sensitive. Would not submit to having her teeth prepared for anything but plastic fillings heretofore, as the pain of excavating, etc., was unbearable. I notified her that I might possibly—by the use of this remedy—be able to operate upon her teeth without pain. She responded and reluctantly agreed to try the experiment. After the dam was adjusted, I applied the cocaine to three approximate cavities in the anterior teeth, and in a few minutes began to excavate. At first they were still tender, but with care and repeated applications, I finally succeeded in preparing all to my entire satisfaction—and comparatively no pain to the patient.

In the following case I obtained the most satisfactory result, and believe this class of cases the most available for relief.

Exp. 3. A large cavity on labial surface of superior cuspid extending high up under gum. Had been filled repeatedly with plastics by various operators, and a number of attempts made to secure free access to entire cavity, every attempt proving painfully unbearable, the operations were abandoned and plastics used. I applied cocaine to the gum surrounding the tooth, and extended the application quite beyond on each side. In a few minutes the parts were anæsthetized; I made another application and proceeded to adjust the dam, and with a clamp forced the gum w–a-y up, exposed and secured the cavity to full view, *and no pain.* Of course, the operation was completed without any other difficulty.

I have tried the cocaine in many other cases with a variety of results; in the aggregate I have had a large per cent of success. The above reported cases I regard as extreme ones, and sufficiently convincing to give this remedy a prominent place in the operating room of the dentist.

The solution used was Merck's, 4 p. c., which decomposes in a few weeks, to avoid which, I append an extract from Nov. "Ephemeris," by Dr. Squibb:

"The solution of Merck's hydrochlorate in distilled water, and the solution filtered, remains entirely clear for two or three weeks; but some that is now a month old shows the usual signs of microscopic growths. But a solution made at the same time, wherein half the water was replaced by a cold saturated solution of salicylic acid, remains entirely clear and bright, and, judging by analogy and experience with the salts of other alkaloids, it will remain clear indefinitely. The solution so protected has been frequently used without discoverable irritation, and from these circumstances it follows that all solutions of salts of cocaine should be so protected from change."

Yours, etc.,

OTTO ARNOLD.

---

IT appears that Russia is to have a dental journal, its first one, too, to be issued from St. Petersburg, and dating from the first of the year. It is to be hoped that the imperial asses in power, in their fear of dynamite, *nitro*-glycerine, and other explosives, may not mistake *nitrous* oxide for something dangerous, hang the editor, banish his family, raze his house, and relegate the journal to the already long list of proscribed books and periodicals.

### THE DENTAL CHAIR.

I love it, I love it, and who shall dare
To chide me for loving that dental chair?
As a child I have sat in it oft for a prize,
I've bedewed it with tears, embalmed it with sighs;
And as since, I've been soothed by the dental art,
'Tis bound by many a plug to my heart.
Would ye learn the spell? A good dentist was there,
And he cured my ache in that dental chair.

In my childhood's hour I lingered by
That velvety seat with wondering eye,
And gazed on the many-pronged teeth that fell
From the mouth of a mother that loved me well.
She told me those pains should never be mine
If I breathed that good dentist's anodyne;
For the grandest legacy man can bequeath
Is a painless mode of extracting teeth.

From that glad time I watched him day by day,
And his eye was bright though his hair was gray;
And still, as the seasons onward rolled,
His teeth would glisten in pearl and gold.
But the years passed on—as the last one sped
My hopes were all shattered--my dentist dead.
And I learned life's keenest pangs to bear
As a bungler profaned that dental chair.

But once more has come the sweet solace of youth,
No longer I dread the decay of a tooth;
The grim fears that harassed my soul have fled,
I've a dentist as good as the one that is dead.
His amalgam, and gold-and-platina alloy,
Fill my teeth to perfection, my heart fills with joy.
My rapture of spirit, oh, who can declare,
As I sit with delight in that old dental chair.—J. W. H.

The above was clipped from some dental publication several years ago, which one, we have now forgotten.

---

DR. JERRY ROBINSON'S Carbolate of Potash is a most excellent remedy for the treatment of suppuration of the gums.

### SERIOUS EFFECTS OF LAUGHING GAS.

WINDFALL, IND., December 26, 1884.—Miss Mollie Lawson, of this place, went to Kokomo last Saturday to have a tooth extracted. Dr. Kirk, the dental surgeon, administered laughing gas to her and pulled the tooth. Shortly afterward she took hemorrhage of the lungs, was taken to the hotel, where she soon became unconscious and went into spasms, and has been having convulsions ever since at intervals of an hour or so, and has remained unconscious almost continually since she was first taken. The attending physicians pronounce her case very critical and uncertain as to her recovery. Yesterday evening her father writes to friends here that she can not live but a short time. She is about twenty years of age, and an estimable young lady.—*Commercial Gazette.*

The above paragraph is rather startling to those who believe that nitrous oxide is innocent of having produced death. In this case there must have existed some lesion that needed but an exciting cause to immediately develop trouble, and this the oxide did, no doubt, very much to Dr. Kirk's surprise.

---

IT has been suggested several times during the past few years (and by very prominent dentists, too,) that the Mississippi Valley Association of Dental Surgeons divest itself of its local character and become what its name really implies. In other words, the society should not hold two meetings in succession in the same city. There are a number of cities in which meetings could be held with great advantage to the society and to the profession of the surrounding country. St. Louis, Louisville, Lexington, Nashville, Memphis, and Vicksburg are all in the Mississippi Valley, and the dentists residing in those cities would, no doubt, be glad to have the society make them a visit in turn, and do all they could to make things comfortable and convenient for attending members. By visiting these localities a much larger attendance would be attracted from the Southern and Southwestern States than at present obtains and would be the means, very likely, of infusing new blood and vigor into the veins of the venerable old lady (we say this with all due respect). In all likelihood it will be well for members to consider this matter and bring the subject up for discussion at the coming meeting and try the experiment again, for, be it remembered, the society was in its earlier days of a migratory habit but could not resist the temptation to settle down in the Queen of the West.

---

THE coming meeting of the Mississippi Valley Dental Society on the 4th of next month, gives evidence, even at this early date, of being large, enthusiastic and profitable. The writer, in the capacity of Chairman of the Executive Committee, has written to a number of dentists throughout the Mississippi Valley requesting a short paper on some topic of interest to the profession and already has the promise of papers from several prominent and well-known writers. This fact will add greatly to the value of the meeting and will induce a large attendance of dentists from the neighboring States. The programme appears upon another page of this issue of the REGISTER, its topics covering sufficient ground to give all shades of opinion an opportunity of expression. The REGISTER will as usual send its reporter to the meeting and publish the proceedings and discussions in the April number.

The officers of the society extend a very cordial invitation to all dentists to be present, and those who are *not* members to become so forthwith, for they cannot do honor and credit to themselves better than by enrolling their names upon the books of the oldest dental society in existence.

---

CAULK'S DENTAL ANNUAL for 1884–85, is at hand and right welcome it is, for it seems like an old friend, so often have we consulted the first issue for various matters. The Annual is greatly enlarged and far more complete than that of last year. The lists of dentists, dealers, and societies are very nearly complete and are quite valuable. It might have been stated that the Ohio State Dental Society was re-organized and *incorporated*, a fact that was not true of the old society. In addition to statistics, it contains a few short and interesting papers and a number of choice tid-bits. Taken altogether, the Annual is of great value to the dentist and every one should contribute his mite towards making its contents still more various and valuable and his twenty-five cents to place his name upon the subscription list.

---

A NUMBER of observers and experimenters writing about muriate of cocaine have stated that no drug has created such a stir and excitement in so short a time; one month from the time of the discovery of its anæsthetic properties, when applied to the eye and the mucous surfaces, every reader in the medical world knew more or less about it, and in turn became an experimenter. There is another side to this, *viz.*: The energy and push of editors and publishers of professional periodicals, their desire to keep abreast of the times and their actual accomplishment of that desire, and yet there are thousands who rarely, if ever, invest a dollar in subscriptions toward encouraging and sustaining the professional press, that great aorta through which the stream of progress is constantly rushing.

---

GRAFTON, W. Va., January 23.--Captain Bankhead, of Virginia, who was conducting a survey of the Grafton and Greenbrier Railroad extension, died at Philippi Tuesday night, by strangulation. He was confined to his bed by sickness, and while under the influence of chloroform, which had been administered to relieve intense pain during the performance of a delicate operation by physicians, a set of false teeth which he wore became loose in his mouth and he strangled to death. He was about sixty years of age.—*Cincinnati Commercial Gazette.*

---

MARRIED--KEELY RHODEHAMEL.--At the residence of the bride's parents on Green Street, Piqua, O., January 14th, 1885, Dr. Charles I. Keely, of Hamilton, and Miss Ella Rhodehamel.

Dr. C. I. Keely is son to Dr. George W. Keely, of Oxford, O. with whom nearly every dentist in the West is acquainted. We extend to the *younger* Dr. Keely (for the father is young) the sincere congratulation of a friend and classmate, and we feel assured that one and all wish him and his bride long life and good health.

AN Englishman, Frenchman, and American were discussing the merits of painters of their respective countries. The American, after listening to all the others had to advance in favor of their countrymen, remarked: "Wall, yes, I guess they did some tall painting, but there was a young fellow in our village, and he got a piece of marble and painted it like cork, and darn me if it didn't float."

---

AN oldest inhabitant was once asked if he had ever experienced a severer snow-storm than that at present raging. "No, I have not," he replied, "and I have resided in Colorado over thirty years." Overhearing this remark, Jupiter, after expressing much surprise, ordered a golden harp and a front seat for the oldest inhabitant.

---

COLUMBIA UNIVERSITY, Washington, D. C., has at last concluded to admit women to its medical classes. Previous to this action, latter part of December, 1884, the women were compelled to go to Philadelphia or Ann Arbor, the latter university having admitted women to its classes ever since its establishment.

---

WE want to see all the readers of the REGISTER at the meeting of the Mississippi Valley Society next month, and want them all to say something good, for we intend to make a good report.

---

LINNÆUS informs us that, by eating plentifully of strawberries every day, he kept himself free from gout. They promote perspiration and dissolve the tartarous incrustations on the teeth.

---

WE stated last month that "muriate of cocaine is worth *only* $180 an ounce." This is a grevious error, the exact price being $480 for the same amount.

---

PROFESSIONAL men ought never to compete in anything save excellence.—*E. T. Darby.*

